# Characteristics and treatment patterns of patients with asthma on multiple-inhaler triple therapy in Spain

**DOI:** 10.1038/s41533-022-00270-2

**Published:** 2022-03-10

**Authors:** Miriam Barrecheguren, Monica Monteagudo, Marc Miravitlles, Xavier Flor, Alexa Núñez, Jeisson Osorio, Xavier Muñoz, Iñigo Ojanguren

**Affiliations:** 1 Department of Pneumology, Hospital Universitari Vall d’Hebron, Vall d’Hebron Institut de Recerca (VHIR), Vall d’Hebron Barcelona Hospital Campus, Barcelona, Spain; 2 Ciber de Enfermedades Respiratorias (CIBERES), Barcelona, Spain; 3 Institut Universitari d’Investigació en Atenció Primària (IDIAP) Jordi Gol, Barcelona, Spain; 4 Centre d’Atenció Primària (CAP) Chafarinas, Institut Català de la Salut (ICS), Barcelona, Spain; 5 Department of Medicine, Universidad Autónoma de Barcelona, Barcelona, Spain; 6 Department of Pulmonary Medicine, Hospital Clínic-Institut d’Investigacions Biomèdiques August Pi i Sunyer (IDIBAPS), University of Barcelona, Barcelona, Spain

**Keywords:** Asthma, Diseases

## Abstract

The aim of this observational, retrospective study was to describe characteristics, treatment patterns, and adherence among patients with asthma who initiated multiple-inhaler triple therapy (MITT) in Catalonia, Spain. This study used data of patients initiating MITT in 2016 from the SIDIAP (Information System for Research in Primary Care) database, which covers ~80% of the Catalonian population (5.8 million). Of 1,204 patients initiating MITT, 361 (30.0%) stepped down (discontinued ≥ 1 and continued ≥1 MITT component) and 89 (7.4%) stopped all three components of MITT for a period of 60 days during the following 12 months. In the follow-up period, 196 (16.3%) patients were considered adherent to MITT (>0.8 proportion of days covered [PDC]), with a mean (standard deviation) PDC of 0.52 (0.51) days. Given the low adherence and substantial rates of step down/discontinuation among patients initiating MITT, there is an urgent need to implement strategies to improve treatment adherence/persistence.

## Introduction

Asthma is a heterogeneous chronic inflammatory respiratory disease that is often both over- and underdiagnosed^1–3^. From 1990 to 2015, the prevalence of asthma increased by 12.6% globally, with over 358 million people worldwide suffering from asthma in 2015^4^. Prevalence rates vary significantly between countries or even between regions of the same country, with the prevalence rate in Spain estimated to be between 2.5 and 13.5% overall, and the prevalence in Barcelona reported to be 6.3–6.6%^5–7^.

Between 30 and 50% of patients with moderate/severe asthma have inadequately controlled disease despite attempts to optimize adherence to inhaled corticosteroids in combination with a long-acting β_2_-agonist (ICS/LABA)^8–12^. In an observational, cross-sectional study conducted in Spain, the prevalence of uncontrolled severe persistent asthma among patients with asthma seen at pneumology and allergology hospital units based on a physician’s diagnosis was 3.9% (*N* = 36,649). Furthermore, the level of asthma control was shown to be underestimated by physicians, which may reflect issues with adopting and implementing treatment guidelines in clinical practice^13^. The most common treatments recommended for both moderate/severe asthma and uncontrolled severe persistent asthma are ICS/LABA alone or in combination with a short-acting β_2_-agonist (SABA) or a leukotriene receptor antagonist^13^. The Global Initiative for Asthma (GINA) also recommends adding long-acting muscarinic antagonists (LAMA) as an additional controller for patients with uncontrolled asthma on at least medium-dose ICS/LABA therapy^2^. The addition of a LAMA to ICS/LABA maintenance therapy as part of a multiple-inhaler triple therapy (MITT) regimen^14^ or, more recently, as single-inhaler triple therapy (SITT) has been shown to improve lung function and reduce exacerbation rates in patients with uncontrolled asthma^15–17^. These data indicate that escalation to triple therapy (ICS/LABA/LAMA) may benefit patients on ICS/LABA with uncontrolled asthma.

The use of multiple inhalers as dual therapy in asthma is associated with lower adherence rates, lower medication persistence, increased asthma-related symptoms, and increased hospitalization compared with single-inhaler dual therapy^18,19^. Currently, there is limited real-world data regarding demographics, clinical characteristics, treatment patterns, discontinuation, and adherence of patients with asthma initiating MITT in Europe. Therefore, the aim of this study was to describe the characteristics of patients with asthma who initiate MITT, treatment patterns before and after MITT initiation, and treatment adherence to MITT in Catalonia, Spain.

## Method

### Study design

This epidemiological study with retrospective analysis of longitudinal follow-up data used the SIDIAP database, which contains anonymized electronic patient records for 5.8 million people registered in 279 primary care centers of the Catalan Health Institute and covers approximately 80% of the population of Catalonia^20^. The SIDIAP database contains information on patient demographics and medical records from 2006. Additionally, the SIDIAP is linked to registers or databases that provide information regarding hospitalizations, medication prescribed in pharmacies within the Catalan Health Institute hospitals (Pharmacy Register), and mortality (National Mortality Register).

**Fig. 1:**
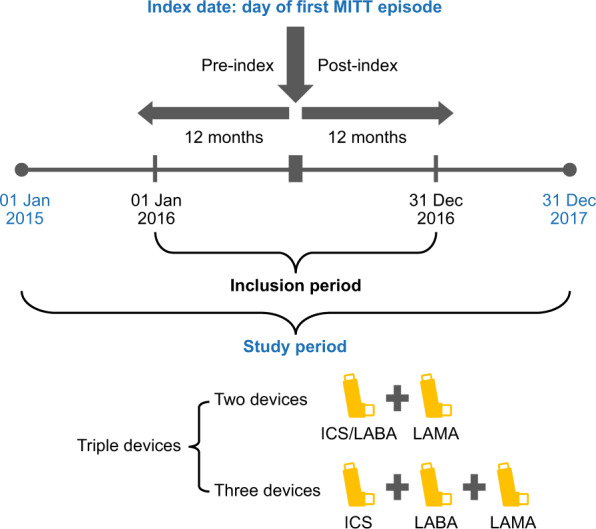
Study design. ICS inhaled corticosteroid, LABA long-acting β_2_-agonist, LAMA long-acting muscarinic antagonist, MITT multiple-inhaler triple therapy.

Patients initiating MITT were identified from January 1st to December 31st, 2016. The index date was the date of initiation of the third component of MITT, defined as overlapping prescriptions for ICS, LABA, and LAMA (in two or three devices) for ≥60 days (Fig. 1). Data were collected for the 12 months prior to (pre-index) and 12 months following (post-index) the index date.

### Study population

Patients included in this study were aged 18–75 years with at least one protocol-defined ICD 10-CM (International Classification of Diseases, Tenth Revision, Clinical Modification^21^) asthma medical code, an overlapping prescription of at least 60 days for each of the three components of MITT during the study inclusion period, and available medical records in the SIDIAP database pre- and post-index.

Patients were excluded if they had a diagnosis of COPD (including emphysema and chronic bronchitis), cystic fibrosis, heart failure, or lung cancer at any time in their medical records, or had used MITT or LAMA monotherapy pre-index (to ensure exclusion of patients with COPD incorrectly classified as having asthma).

### Study endpoints

The primary objective of the study was to describe the demographics, comorbidities, clinical characteristics, and treatment patterns in the overall study population pre- and post-index.

The secondary objectives were to describe discontinuation of MITT post-index (including the number of patients discontinuing and the number of patients stepping down and subsequent treatment patterns); describe patient characteristics according to MITT persistence or discontinuation post-index; describe the characteristics and treatment patterns of patients who experienced severe exacerbations pre-index; to describe treatment adherence in patients on MITT post-index, and to describe characteristics and treatment patterns in patients who discontinued MITT according to the timing of discontinuation (first 90 days or Days 181–364).

Three different MITT treatment patterns were assessed post-index: persistence on MITT; step down from MITT; and discontinuation of MITT. Persistence on MITT included patients who did not discontinue and did not step down; step down from MITT included patients who discontinued at least one of the three MITT components but continued to be prescribed at least one component; and discontinuation of MITT included patients who experienced a 60-day gap in subsequent prescriptions for all three components (i.e., ICS, LABA, LAMA). The rationale of using a 60-day gap to define discontinuation was based on requirements of the healthcare system in Catalonia where regular prescription of inhaled therapies by clinicians lasts for 2 months; after 2 months it is mandatory for the physician to renew or modify the prescription. The date of step down or discontinuation was defined as the last date of overlapping prescription of ICS, LABA, and LAMA.

### Outcomes

Demographics, comorbidities, and clinical characteristics including lung function (FEV_1_ % predicted value and FEV_1_/FVC ratio), the percentage of eosinophils in peripheral blood, severe exacerbations, and treatments patterns were identified from the SIDIAP database pre- and post-index. Severe exacerbations were defined as the need for one hospitalization or emergency visit or the use of oral/systemic corticosteroids (or increase in the maintenance dose) for ≥3 days due to asthma, and were captured by the Catalan Health Institute hospitals. Additional outcomes assessed post-index included adherence to MITT, defined as the extent to which a patient acts in accordance with the prescribed interval and dose of a dosing regimen. In this study, treatment adherence was assessed in terms of PDC.

### Sample size and statistical analysis

All patients in the SIDIAP registry (~5.8 million people) who met the eligibility criteria were included in the study. Based on an estimated asthma prevalence of 3% in Spain, a sample size of 1,117 patients was considered to be sufficient to address the main study objective with a 95% CI. For patients who discontinued MITT, assuming a 95% CI in a two-sided test, 195 patients in each group (discontinued or not discontinued) were sufficient to find statistically significant differences with a minimum statistical power of 80%.

Continuous variables are reported as a mean and SD and categorical variables are presented as number and percentage of patients. For spirometry data, paired analyses (when available) for a small subgroup of patients are presented.

MITT discontinuation was calculated based on the number of patients who discontinued treatment in relation to the total number of patients in the 12 months pre-index date.

Adherence to MITT was calculated according to the number of adherent patients in relation to the total number of patients in the 12 months post-index date. PDC was calculated during the 12-month post-index period by dividing the total number of days that MITT was prescribed by the number of days post-index and ranged from 0 to 1. A patient was considered adherent when the PDC value was >0.8.

A logistic regression model was developed to identify independent factors associated with adherence to and discontinuation of MITT. The Hosmer–Lemeshow test^22^ was used and independent variables were age, sex, BMI, smoking status, comorbidities (pneumonia, sleep apnea syndrome, polyposis, allergic rhinitis, atopic dermatitis, conjunctivitis, gastroesophageal reflux, diabetes mellitus, hypertension, anxiety, depression), exacerbations, eosinophil count, chest X-ray, computerized tomography scan, allergy test, GP, nurse and pulmonologist visits, pneumologist and allergy referrals, sick leave, sick leave due to a respiratory cause, influenza and pneumococcal vaccines, and previous treatment (ICS, ICS/LABA, ICS/LABA/anti-leukotrienes, ICS/LABA/OCS/anti-leukotrienes, ICS/LAMA, LAMA, LABA/LAMA, LAMA/anti-leukotrienes, anti-leukotrienes, OCS, no treatment). Adherence to MITT refers to the collection of over 80% of the prescribed treatment from pharmacies, whereas discontinuation refers to not having a prescription renewal by the doctor. Analyses were performed using the R (version 3.3.2) statistical package. For statistical tests performed with the outcome variables, a significance level of 0.05 was used, with no adjustments for multiplicity.

## Results

### Characteristics and treatment patterns pre- and post-index

**Fig. 2:**
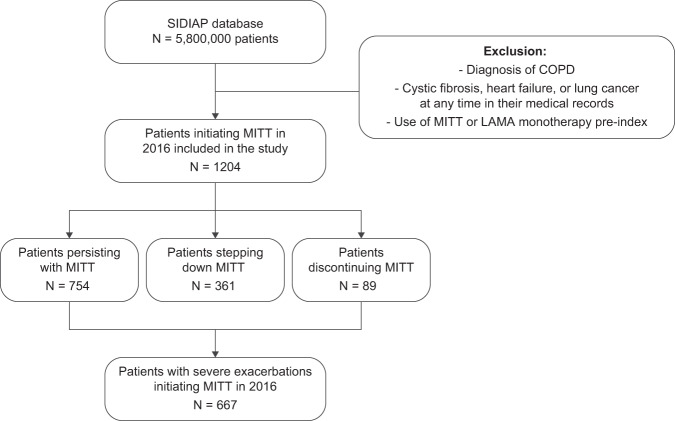
Flow chart of the patient selection from the SIDIAP database. COPD chronic obstructive pulmonary disease, LAMA long-acting muscarinic antagonist, MITT multiple-inhaler triple therapy, SIDIAP Information System for Research in Primary Care.

**Fig. 3:**
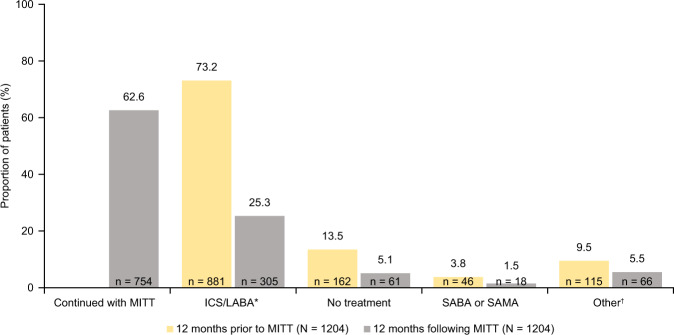
Treatment patterns 12 months prior to (pre-index) and 12 months following (post-index) MITT initiation in the overall population. Percentages may not total 100% due to rounding. *ICS/LABA category includes: ICS/LABA, ICS/LABA/anti-leukotrienes, ICS/LABA/OCS, ICS/LABA/OCS/SAMA, ICS/LABA/anti-leukotrienes/OCS; ^†^other category includes: ICS, ICS/LAMA, ICS/anti-leukotrienes, ICS/OCS, ICS/OCS/LAMA, LABA/LAMA, LABA/anti-leukotrienes, LAMA, LAMA/anti-leukotrienes, anti-leukotrienes, OCS, OCS/LAMA, OCS/LAMA/anti-leukotrienes. ICS inhaled corticosteroid, LABA long-acting β_2_-agonist, LAMA long-acting muscarinic antagonist, MITT multiple-inhaler triple therapy, OCS oral corticosteroid, SABA short-acting β_2_-agonist, SAMA short-acting muscarinic antagonist.

Details of patient selection from the Information System for Research in Primary Care (SIDIAP) database are summarized in Fig. 2. Of 1,204 patients included in this study, 851 (70.7%) were female and the mean (standard deviation [SD]) age at MITT initiation was 54.8 (13.8) years (Table 1). Body mass index (BMI), smoking status, and percentage of eosinophils measured in peripheral blood were comparable pre- and post-index (Table 1). Mean (SD) forced expiratory volume in 1 s (FEV_1_) % predicted was 70.8 (19.1) pre-index and 75.4 (19.6) post-index (Table 1). Among the 1,204 patients included, 881 (73.2%) had previously been receiving ICS/LABA and 162 (13.5%) were not receiving any treatment (Fig. 3).

**Table 1:** Demographics and clinical characteristics 12 months prior to and 12 months following MITT initiation.

	12 months prior to MITT (pre-index) (*N* = 1204)	12 months following MITT (post-index) (*N* = 1204)
Demographics		
Female, *n* (%)	851 (70.7)	
Age at asthma diagnosis, mean (SD)	45.2 (15.9)	
Age at MITT initiation, mean (SD)	54.8 (13.8)	
Years from diagnosis to MITT, mean (SD)	9.6 (8.9)	
Urban location, *n* (%)	1,023 (85.0)	
Smoking status, *n* (%)		
Non-smoker^a^	708 (58.8)	717 (59.6)
Smoker	208 (17.3)	199 (16.5)
Former smoker	247 (20.5)	272 (22.6)
Unknown	41 (3.4)	16 (1.3)
BMI^b^	*n* = 640	*n* = 536
BMI, mean (SD) kg/m^2^	30.3 (6.2)	30.9 (6.3)
Spirometry^c^, mean (SD)	*n* = 387	*n* = 281
FEV_1_ % predicted value	70.8 (19.1)	75.4 (19.6)
FEV_1_/FVC ratio	70.1 (13.1)	73.6 (13.3)
Common comorbidities, *n* (%)		
Anxiety/depression	459 (38.1)	
Pneumonia	57 (4.7)	
Other respiratory infections	373 (31)	
Rhinitis	232 (19.3)	
Hypertension	409 (34.0)	
Diabetes mellitus	139 (11.5)	
Gastroesophageal reflux	112 (9.3)	
Conjunctivitis	12 (10.0)	
Polyposis	76 (6.3)	
Severe exacerbations^d^; *n* (%)	667 (55.4)	503 (41.8)
Blood eosinophils (%), mean (SD)	*n* = 732 4.1 (3.4)	*n* = 717 4.1 (3.1)

*BMI* body mass index, *FEV*_*1*_ forced expiratory volume in 1 s, *FVC* forced vital capacity, *SD* standard deviation, *MITT* multiple-inhaler triple therapy.

^a^The number of non-smokers is higher following MITT initiation as the smoking status for some patients only became available during that period;

^b^reported for patients with a BMI record in their medical history;

^c^reported for patients with spirometry measurements in their medical history;

^d^a severe exacerbation was defined as the need for one hospitalization or emergency visit or the use of oral/systemic corticosteroids (or increase in the maintenance dose) for at least 3 days due to asthma.

### Patients stepping down and discontinuing MITT and subsequent treatments

**Fig. 4:**
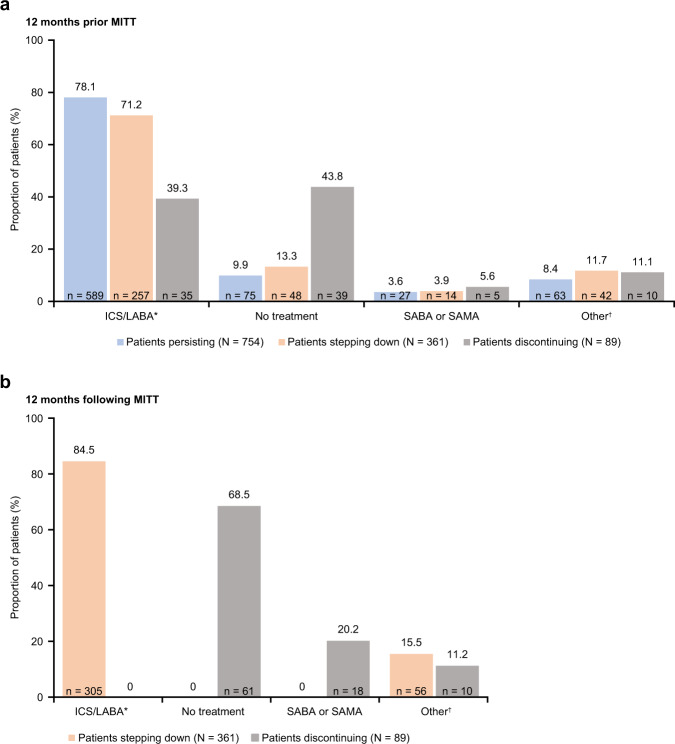
Treatment patterns in patients that persist, step-down and discontinue MITT. **a** 12 months prior to (pre-index) and **b** 12 months following (post-index) MITT initiation. Percentages may not total 100% due to rounding. *ICS/LABA category includes: ICS/LABA, ICS/LABA/anti-leukotrienes, ICS/LABA/OCS, ICS/LABA/OCS/SAMA, ICS/LABA/anti-leukotrienes/OCS; ^†^other category includes: ICS, ICS/LAMA, ICS/anti-leukotrienes, ICS/OCS, ICS/OCS/LAMA, LABA/LAMA, LABA/anti-leukotrienes, LAMA, LAMA/anti-leukotrienes, anti-leukotrienes, OCS, OCS/LAMA, OCS/LAMA/anti-leukotrienes. ICS inhaled corticosteroid, LABA long-acting β_2_-agonist, LAMA long-acting muscarinic antagonist, MITT multiple-inhaler triple therapy, OCS oral corticosteroid, SABA short-acting β_2_-agonist, SAMA short-acting muscarinic antagonist.

Among the patients that initiated MITT, 754 (62.6%) persisted with MITT, 361 (30.0%) stepped down (discontinued ≥ 1 and continued ≥1 MITT component) and 89 (7.4%) discontinued MITT (stopped all three components of MITT for a period of 60 days) during the following 12 months. During the pre-index period, 589 (78.1%) patients who persisted with MITT, 257 (71.2%) who stepped down and 35 (39.3%) who discontinued MITT were on ICS/LABA (Fig. 4a). During the post-index period, among the patients that stepped down, 305 (84.5%) were on ICS/LABA and, of the patients that discontinued MITT completely, 61 (68.5%) were not treated and 18 (20.2%) were receiving treatment with SABA or SAMA (Fig. 4b).

There were no clinically relevant differences between patients that stepped down or discontinued MITT compared with those who persisted in terms of age, BMI, or smoking status pre-index (Table 2) and post-index (Supplementary Table 1). Patients who persisted with MITT had similar FEV_1_ % predicted value and mean FEV_1_/forced vital capacity (FVC) ratio pre-index (Table 2) and post-index (Supplementary Table 1) compared with patients who did not maintain MITT. Additionally, in patients who discontinued treatment, the mean (SD) percentage of eosinophils measured in peripheral blood post-index was lower (3.2% [2.0]) compared with patients who persisted (4.0% [3.2]) or stepped down (4.4% [3.0]) from MITT (Supplementary Table 1). Prior to MITT initiation, the percentage of patients experiencing severe exacerbations was similar in patients who persisted, stepped down, or discontinued MITT (55.8% vs 55.1% and 52.8%, respectively) (Table 2).

**Table 2:** Demographics and clinical characteristics 12 months prior to MITT initiation by MITT persistence.

	MITT persistence (*N* = 754)	MITT step down (*N* = 361)	MITT discontinuation (*N* = 89)
Demographics			
Female, *n* (%)	513 (68.0)	273 (75.6)	65 (73.0)
Age at asthma diagnosis, mean (SD)	45.13 (15.6)	45.4 (16.3)	45.37 (16.2)
Age at MITT initiation, mean (SD)	55.30 (13.2)	54.0 (14.5)	54.25 (15.2)
Years from diagnosis to MITT, mean (SD)	10.17 (9.2)	8.6 (8.2)	8.88 (8.5)
Urban location, *n* (%)	642 (85.1)	309 (85.6)	72 (80.9)
Smoking status, *n* (%)			
Non-smoker	439 (58.2)	218 (60.4)	51 (57.3)
Smoker	134 (17.8)	52 (14.4)	22 (24.7)
Former smoker	153 (20.3)	79 (21.9)	15 (16.9)
Unknown	28 (3.7)	12 (3.3)	1 (1.1)
BMI^a^	*n* = 388	*n* = 213	*n* = 39
BMI, mean (SD) kg/m^2^	30.3 (6.1)	30.2 (6.3)	30.0 (6.4)
Spirometry^b^ mean (SD)	*n* = 244	*n* = 124	*n* = 19
FEV_1_ % predicted value	69.4 (18.9)	72.9 (19.0)	74.2 (21.2)
FEV_1_/FVC ratio	69.1 (13.1)	71.5 (12.6)	74.5 (14.7)
Common comorbidities, *n* (%)			
Anxiety/depression	281 (37.3)	150 (41.6)	28 (31.5)
Pneumonia	42 (5.6)	11 (3.0)	4 (4.5)
Other respiratory infections	225 (29.8)	118 (32.7)	30 (33.7)
Rhinitis	142 (18.8)	74 (20.5)	16 (18.0)
Hypertension	254 (33.7)	125 (34.6)	30 (33.7)
Diabetes mellitus	90 (11.9)	41 (11.4)	8 (9.0)
Gastroesophageal reflux	71 (9.4)	36 (10.0)	5 (5.6)
Conjunctivitis	67 (8.9)	44 (12.2)	10 (11.2)
Polyposis	53 (7.0)	21 (5.8)	2 (2.2)
Severe exacerbations^c^, *n* (%)	421 (55.8)	199 (55.1)	47 (52.8)
Blood eosinophils (%), mean (SD)	*n* = 453 4.0 (3.5)	*n* = 232 4.4 (3.4)	*n* = 47 3.0 (1.9)

*BMI* body mass index, *FEV*_*1*_ forced expiratory volume in 1 s, *FVC* forced vital capacity, *SD* standard deviation, *MITT* multiple-inhaler triple therapy.

^a^Reported for patients with a BMI record in their medical history;

^b^reported for patients with spirometry measurements in their medical history;

^c^a severe exacerbation was defined as the need for one hospitalization or emergency visit or the use of oral/systemic corticosteroids (or increase in the maintenance dose) for ≥3 days due to asthma.

Demographics, clinical characteristics, and treatment patterns were generally similar across patients that stopped MITT in the first 90 days (Supplementary Table 2) and those who stopped between Days 181–364 (Supplementary Table 3).

### Characteristics and treatment patterns of patients who experienced severe exacerbations during the 12 months prior to MITT initiation

**Fig. 5:**
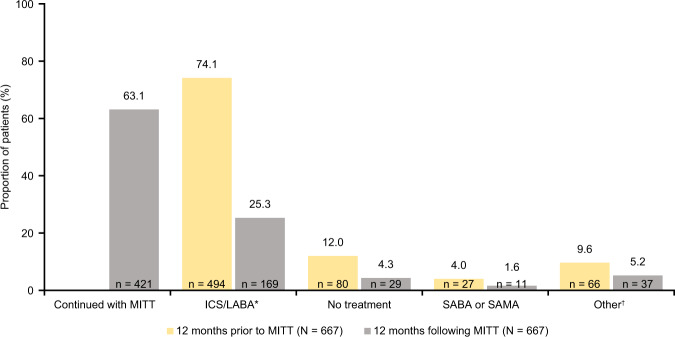
Treatment patterns in the 12 months prior to (pre-index) and 12 months following (post-index) MITT initiation among patients with severe exacerbations. Percentages may not total 100% due to rounding. *ICS/LABA category includes: ICS/LABA, ICS/LABA/anti-leukotrienes, ICS/LABA/OCS, ICS/LABA/OCS/SAMA, ICS/LABA/anti-leukotrienes/OCS; ^†^other category includes: ICS, ICS/LAMA, ICS/anti-leukotrienes, ICS/OCS, ICS/OCS/LAMA, LABA/LAMA, LABA/anti-leukotrienes, LAMA, LAMA/anti-leukotrienes, anti-leukotrienes, OCS, OCS/LAMA, OCS/LAMA/anti-leukotrienes. ICS inhaled corticosteroid, LABA long-acting β_2_-agonist, LAMA long-acting muscarinic antagonist, MITT multiple-inhaler triple therapy, OCS oral corticosteroid, SABA short-acting β_2_-agonist, SAMA short acting muscarinic antagonist.

In the pre-index period, 667 (55.4%) patients experienced at least one severe exacerbation compared with 503 (41.8%) patients post-index (Table 1). Among the patients who experienced severe exacerbations prior to initiation of MITT, 491 (73.6%) were female with a mean (SD) age at asthma diagnosis of 45.4 (15.8) years and a mean (SD) BMI of 30.4 (6.4) kg/m^2^ (Table 3). Additionally, post-index, 352 (52.8%) patients in this subgroup experienced a severe exacerbation. There was no change in the percentage of eosinophils measured in peripheral blood prior to and following MITT initiation (Table 3). Prior to MITT, 494 (74.1%) patients were previously treated with ICS/LABA and 80 (12.0%) patients received no treatment (Fig. 5). Post-index, 169 (25.3%) patients received ICS/LABA and 29 (4.3%) patients did not receive any treatment (Fig. 5).

**Table 3:** Characteristics of patients with severe exacerbations in the 12 months prior to MITT initiation and 12 months following MITT initiation.

	12 months prior to MITT (pre index) (*N* = 667)	12 months following MITT (post index) (*N* = 667)
Demographics		
Female, *n* (%)	491 (73.6)	
Age at asthma diagnosis, mean (SD)	45.4 (15.8)	
Age at MITT initiation, mean (SD)	55.0 (13.8)	
Years from diagnosis to MITT, mean (SD)	9.6 (8.5)	
Urban location, *n* (%)	573 (85.9)	
Smoking status, *n* (%)		
Non-smoker^a^	412 (61.8)	414 (62.1)
Smoker	109 (16.3)	102 (15.3)
Former smoker	131 (19.6)	146 (21.9)
Unknown	15 (2.2)	5 (0.7)
BMI^b^	*n* = 376	*n* = 302
BMI, mean (SD) kg/m^2^	30.4 (6.4)	30.8 (6.4)
Spirometry^c^, mean (SD)	*n* = 215	*n* = 135
FEV_1_ % predicted value	70.6 (19.1)	74.7 (20.1)
FEV_1_/FVC ratio	69.6 (13.6)	73.8 (13.5)
Common comorbidities, *n* (%)		
Anxiety/depression	272 (40.8)	
Pneumonia	32 (4.8)	
Other respiratory infections	233 (34.9)	
Rhinitis	131 (19.6)	
Hypertension	243 (36.4)	
Diabetes mellitus	79 (11.8)	
Gastroesophageal reflux	72 (10.8)	
Conjunctivitis	77 (11.5)	
Polyposis	51 (7.6)	
Severe exacerbations^d^, *n* (%)	667 (100.0)	352 (52.8)
Blood eosinophils (%), mean (SD)	*n* = 425 4.1 (3.6)	*n* = 417 4.1 (3.3)

*BMI* body mass index, *FEV*_*1*_ forced expiratory volume in 1 s, *FVC* forced vital capacity, *SD* standard deviation, *MITT* multiple-inhaler triple therapy.

^a^The number of non-smokers is higher following MITT initiation as for some patients, the smoking status only became available during that period;

^b^reported for patients with a BMI record in their medical history;

^c^reported for patients with spirometry measurements in their medical history;

^d^a severe exacerbation was defined as the need for one hospitalization or emergency visit or the use of oral/systemic corticosteroids (or increase in the maintenance dose) for ≥3 days due to asthma.

### Adherence to MITT: Independent factors associated with adherence and discontinuation, and patient characteristics

Adherence to MITT was assessed using proportion of days covered (PDC), which was calculated as follows: PDC = total days all drugs available/days post-index. A patient was considered adherent when PDC was >0.8.

In the 12 months post-index, 196 (16.3%) patients were adherent to MITT and the mean (SD) PDC was 0.52 (0.51) days. The logistic regression model showed that greater adherence was associated with having anxiety and depression, taking sick leave due to a respiratory cause, and no previous ICS/LABA treatment prior to initiation of MITT (Table 4). Although older age at MITT initiation showed a significant association with greater adherence, the odds ratio was only marginally above 1.0 (1.047; 95% confidence interval [CI]: 1.025–1.070) (Table 4).

**Table 4:** Independent factors associated with adherence to MITT determined by the logistic regression model.

Variable and reference arm	Estimated adjusted OR of adherence to MITT^a^	95% CI	*p*-value in the model
Age at MITT initiation: older vs younger	1.047	1.025–1.070	<0.001
Having anxiety and depression: yes vs no	1.817	1.045–3.159	0.034
Previous sick leave of respiratory cause: yes vs no	2.616	1.235–5.541	0.012
Previous ICS/LABA use: yes vs no	0.528	0.308–0.904	0.020

Adherence to MITT defined as PDC > 0.8.

*BMI* body mass index, *CI* confidence interval, *GP* general practitioner, *ICS* inhaled corticosteroids, *LABA* long-acting β2-agonist, *LAMA* long-acting muscarinic antagonist, *MITT* multiple-inhaler triple therapy, *PDC* proportion of days covered, *OCS* oral corticosteroids, *OR* odds ratio.

^a^Higher OR is associated with a greater likelihood of being adherent to MITT. Independent variables were age, sex, BMI, smoking status, comorbidities (pneumonia, sleep apnea syndrome, polyposis, allergic rhinitis, atopic dermatitis, conjunctivitis, gastroesophageal reflux, diabetes mellitus, hypertension, anxiety, depression), exacerbations, eosinophil count, chest X-ray, computerized tomography scan, allergy test, GP, nurse and pulmonologist visits, pneumologist and allergy referrals, sick leave, sick leave due to a respiratory cause, influenza and pneumococcal vaccines, and previous treatment (ICS, ICS/LABA, ICS/LABA/anti-leukotrienes, ICS/LABA/OCS/anti-leukotrienes, ICS/LAMA, LAMA, LABA/LAMA, LAMA/anti-leukotrienes, anti-leukotrienes, OCS, no treatment).

The proportion of patients who were current smokers and those experiencing a severe exacerbation was higher in adherent patients compared with non-adherent patients, whereas the percentage of eosinophils in peripheral blood was slightly higher in non-adherent versus adherent patients (Supplementary Table 4). Being male, not receiving a previous allergy test, or no previous treatment prior to initiation of MITT were all associated with discontinuation of MITT (Supplementary Table 5). Anxiety/depression was marginally but non-significantly (*p* = 0.052) associated with discontinuation of MITT (OR [95% CI]: 1.562 [0.996–2.448]).

## Discussion

This retrospective observational study analyzed characteristics and treatment patterns of 1,204 patients with asthma initiating MITT in Catalonia, Spain. Overall, while persistence rates were relatively high, the rates of step down and discontinuation of MITT were also high, and adherence was poor. Most of the patients who initiated MITT during the study period were on ICS/LABA prior to index date, while 13% were untreated. In the 12 months after initiating MITT, 63% of patients persisted on MITT, 30% stepped down and 7% discontinued MITT. The proportion of adherent patients (>0.8 PDC) was only 16%; being older, having anxiety and/or depression, taking sick leave due to respiratory symptoms, and no previous treatment with ICS/LABA prior to initiation of MITT were all associated with greater adherence to treatment.

In our study, the unexpected finding that 14% of patients were not receiving any treatment prior to initiation of MITT could perhaps be attributed to a late diagnosis of severe asthma or a failure of physicians to adhere to guideline recommendations leading to potential overtreatment. Additionally, the high rates of treatment discontinuation/stepping down might be associated with both physician behavior (e.g., justified discontinuation/step-down due to lack of symptom control) and patient behavior (e.g., missed GP appointments, having a prescription but not filling it, and difficulties using multiple inhalers).

Suzuki et al published a similar observational cohort study in patients with asthma and asthma/chronic obstructive pulmonary disease (COPD) overlap who initiated triple therapy in Japan^23^. Persistence rates among patients with asthma were lower than those reported here (38.5% over 12 months)^23^. Furthermore, in our study, most patients (73%) initiating MITT were on ICS/LABA prior to the index date, compared with nearly 100% of patients in the observational study in Japan^23^. However, the sample size was much smaller. Studies on COPD using data from the SIDIAP database used in this study showed that 16.5% of patients initiated triple therapy 1 year after diagnosis^24^, and reported similar persistence rates with 62.5% of patients continuing to receive MITT 12 months following MITT initiation ^25^.

The benefits of initiating triple therapy on lung function, symptoms, and asthma control in patients with uncontrolled asthma have been demonstrated in multiple studies^14–17^. This study was not designed to determine any change in clinical outcomes before and after MITT initiation; however, a reduction in the number of patients experiencing a severe exacerbation was seen in the 12 months following MITT initiation compared with the previous 12 months (55.4 and 41.8% of patients, respectively). A reduction was observed in the aforementioned Japanese study (64.0% vs 45.8%)^23^. Interestingly, in our study, a greater reduction was observed in the subgroup of patients that had a severe exacerbation in the 12 months prior to MITT, with MITT reducing the number of patients experiencing exacerbations by half.

Triple therapy with ICS/LABA plus tiotropium has been shown to improve lung function and reduce the risk of exacerbations in patients with asthma^26^; however, the addition of tiotropium requires the use of multiple inhalers with different dosing regimens^14^. More recently, the TRIMARAN, TRIGGER, CAPTAIN, and IRIDIUM studies^15–17^ evaluated the efficacy and safety of various triple ICS/LABA/LAMA combinations administered via single inhalers. These studies demonstrated favorable effects on asthma clinical and patient-reported outcomes with no new safety concerns^15–17^. Observed differences between these studies (e.g., on asthma exacerbations) were likely due to differences in study populations and outcome definitions used ^27^.

Adherence to therapy is complex and it is influenced by interdependent factors related to the patient, the healthcare provider, and the healthcare system. In Spain, low adherence rates to triple therapy in COPD have been associated with high costs to the Spanish National Health System^28^. In this study, adherence to triple therapy in the 12 months following MITT initiation was 16.3% and the mean (SD) PDC was 0.52 (0.51) days. A similar low adherence rate was reported in the Japanese study discussed previously with a PDC of 0.51 in the asthma-only group as well as in the asthma-COPD overlap group^23^. It has been reported that persistence and adherence rates are lower when using multiple inhalers versus single-inhaler dual therapy in both asthma and COPD^18,28,29^. While the reasons for poor adherence in this study are unknown, possible explanations include the high cost of asthma medications and requirements for copayments (even in publicly funded healthcare systems such as in Spain), lack of patient understanding/awareness, limited supervision, or follow-up with a healthcare professional, lack of awareness of disease severity, and complexities for patients and for caregivers in using multiple inhalers with different dosing regimens (e.g., once vs twice daily)^30–33^. Additionally, an observational study conducted in Australia showed that over 80% of patients stepped-up to GINA Step 5 therapies while showing poor adherence to ICS/LABA^34^. Based on this, further analyses to determine the PDC before initiation of MITT might give important insights into the low adherence rates reported here. Notably, although adherence to MITT was low in this study, most patients (62.6%) persisted on MITT, which may indicate that patients experienced benefits on triple therapy. Nonetheless, the poor adherence rates we observed highlight a need for improvement. Offering SITT could benefit these patients and improve adherence. Interestingly, in this study greater adherence was associated with having anxiety and/or depression, taking sick leave due to a respiratory cause, and no previous ICS/LABA treatment. Studies have shown that treatment adherence tends to be higher among patients with uncontrolled and severe asthma^35,36^. The fact that taking sick leave was associated with higher adherence may indicate that patients in this study were perhaps more symptomatic or perceived their asthma as severe.

Strengths of our study include the large sample size, good representation of the population, and lack of a recall bias as this is a database study. However, as this was an observational retrospective study, it has inherent limitations including under-reporting of exacerbations, miscoding biases, and invalid/unknown data. To mitigate the impact of invalid/unknown data, we have used a range of diagnostic codes that delimited the study disease. Finally, spirometry and blood eosinophil data were limited due to the small number of patients with available data.

Overall, results from this study in patients with asthma initiating MITT showed that a significant proportion of patients step down, discontinue MITT, and are poorly adherent during the first 12 months of treatment. The poor adherence and substantial rates of step down/discontinuation we observed in this study, highlights an urgent need to implement strategies to improve treatment adherence. Addressing factors such as accuracy of diagnosis, inhaler technique, complexity of dosing regimens, and regularly checking treatment adherence in patients currently on ICS/LABA prior to initiating MITT may all be beneficial.

### Reporting summary

Further information on research design is available in the Nature Research Reporting Summary linked to this article.

## Supplementary information

Supplementary Materials

REPORTING SUMMARY

## Data Availability

The data that support the findings of this study belong to the Catalan Healthcare. System and are not publicly available. Anonymised individual participant data and study documents can be requested for further research from www.clinicalstudydatarequest.com.
