# Supplementary material for: Characteristics and treatment patterns of patients with asthma on multiple-inhaler triple therapy in Spain
Source: NPJ Prim Care Respir Med. 2022 Mar 10;32:11. doi: 10.1038/s41533-022-00270-2 (PMC8913688; doi:10.1038/s41533-022-00270-2)
Supplement: Supplementary file 1 — Supplementary Materials [file 41533_2022_270_MOESM1_ESM.pdf]

Supplementary materials

Supplementary Table 1. Characteristics of patients in the 12 months following MITT initiation (post-index) in those that persist, step down or discontinue

	MITT persistence (N=754)	MITT stepdown (N=361)	MITT discontinuation (N=89)
**Smoking status, n (%)**			
Non-smoker	445 (59.0)	221 (61.2)	51 (57.3)
Smoker	131 (17.4)	49 (13.6)	19 (21.3)
Former smoker	168 (22.3)	86 (23.8)	18 (20.2)
Unknown	10 (1.3)	5 (1.4)	1 (1.1)
**BMI**[1]	**n=335**	**n=164**	**n=37**
BMI, mean (SD) kg/m^2	30.9 (6.0)	31.1 (6.7)	30.6 (7.5)
**Spirometry**[2]**, mean (SD)**	**n=185**	**n=80**	**n=16**
FEV_1 % predicted value	73.7 (19.0)	78.3 (20.4)	80.5 (20.1)
FEV_1/FVC ratio	72.0 (13.1)	76.3 (13.4)	78.8 (12.4)
**Severe exacerbations**[3]**, n (%)**	310 (41.1)	158 (43.8)	35 (39.3)
**Blood eosinophils (%), mean (SD)**	**n=448**	**n=217**	**n=53**
	4.0 (3.2)	4.4 (3.0)	3.2 (2.0)

[1]Reported for patients with a BMI record in their medical history; [2]reported for patients with spirometry measurements in their medical history; [3]a severe exacerbation was defined as the need for one hospitalisation or emergency visit or the use of oral/systemic corticosteroids (or increase in the maintenance dose) for ≥3 days due to asthma.

BMI, body mass index; FEV_1, forced expiratory volume in 1 second; FVC, forced vital capacity; MITT, multiple-inhaler triple therapy; SD, standard deviation.

Supplementary Table 2. Characteristics of patients who stopped MITT in the first 90 days and in patients that stopped MITT between Days 181 and 364

	**Patients that stopped MITT in the first 90 days**			**Patients that stopped MITT between Days 181 and 364**		
	**Total N=142**	**Stepping down from MITT N=121**	**Discontinuing MITT N=21**	**Total N=162**	**Stepping down from MITT N=125**	**Discontinuing MITT N=37**
**Demographics**						
Female, n (%)	104 (73.2)	89 (73.6)	15 (71.4)	122 (75.3)	96 (76.8)	26 (70.3)
Age at asthma diagnosis, mean (SD)	43.5 (16.2)	43.3 (16.6)	44.4 (13.9)	45.5 (15.4)	46.4 (15.1)	42.3 (16.1)
Age at MITT initiation, mean (SD)	52.4 (14.9)	51.9 (15.1)	55.5 (13.3)	54.0 (14.2)	54.9 (13.7)	51.2 (53.7)
Years from diagnosis to MITT, mean (SD)	9.0 (8.2)	8.6 (8.3)	11.1 (6.9)	8.5 (8.4)	8.4 (8.2)	8.9 (8.9)
Urban location, n (%)	125 (88.0)	106 (87.6)	19 (90.5)	134 (82.7)	105 (84.0)	29 (78.4)
**Smoking status (prior to MITT), n (%)**						
Non-smoker	93 (65.5)	77 (63.6)	16 (76.2)	92 (56.8)	71 (56.8)	21 (56.8)
Smoker	21 (14.8)	17 (14.0)	4 (19.0)	26 (16.0)	15 (12.0)	11 (29.7)
Former smoker	22 (15.5)	21 (17.4)	1 (4.8)	42 (25.9)	37 (29.6)	5 (13.5)
Unknown	6 (4.2)	6 (5.0)	0 (0.0)	2 (1.2)	2 (1.6)	0 (0.0)
**BMI (prior to MITT)**[1]	**n=77**	**n=69**	**n=8**	**n=88**	**n=75**	**n=13**
BMI, mean (SD) kg/m^2	29.6 (6.2)	29.6 (6.3)	28.9 (5.8)	30.3 (6.2)	30.1 (6.2)	31.2 (6.8)
**Spirometry (prior to MITT)**[2]**, mean (SD)**	**n=6**	**n=6**	**n=0**	**n=14**	**n=13**	**n=1**
FEV_1 % predicted value	70.7 (12.4)	70.7 (12.4)		66.6 (18.8)	66.7 (19.4)	65.7
FEV_1/FVC ratio	68.0 (14.2)	68.0 (14.2)		70.1 (10.1)	70.0 (10.5)	71.5

**Common comorbidities, n (%)**						
Anxiety/depression	53 (37.3)	47 (38.8)	6 (28.6)	65 (40.1)	54 (43.2)	11 (29.7)
Pneumonia	3 (2.1)	3 (2.5)	0 (0.0)	8 (4.9)	6 (4.8)	2 (5.4)
Other respiratory infections	48 (33.8)	39 (32.2)	9 (42.9)	52 (32.1)	43 (34.4)	9 (24.3)
Rhinitis	31 (21.8)	28 (23.1)	3 (14.3)	40 (24.7)	31 (24.8)	9 (24.3)
Hypertension	46 (32.4)	37 (30.6)	9 (42.9)	55 (34.0)	45 (36.0)	10 (27.0)
Diabetes mellitus	18 (12.7)	17 (14.0)	1 (4.8)	18 (11.1)	15 (12.0)	3 (8.1)
Gastroesophageal reflux	15 (10.6)	12 (9.9)	3 (14.3)	13 (8.0)	12 (9.6)	1 (2.7)
Conjunctivitis	22 (15.5)	19 (15.7)	3 (14.3)	19 (11.7)	16 (12.8)	3 (8.1)
Polyposis	6 (4.2)	4 (3.3)	2 (9.5)	6 (3.7)	6 (4.8)	0 (0.0)
**Severe exacerbations[3], n (%)**	84 (59.2)	70 (57.9)	14 (66.7)	88 (54.3)	69 (55.2)	19 (51.4)
**Blood eosinophils (%), mean (SD)**	**n=83**<br>3.9 (3.1)	**n=71**<br>4.0 (3.2)	**n=12**<br>3.2 (1.9)	**n=93**<br>4.1 (2.8)	**n=78**<br>4.3 (2.9)	**n=15**<br>3.1 (2.5)

[1]Reported for patients with a BMI record in their medical history; [2]reported for patients with spirometry measurements in their medical history; [3]a severe exacerbation was defined as the need for one hospitalisation or emergency visit or the use of oral/systemic corticosteroids (or increase in the maintenance dose) for ≥3 days due to asthma.

BMI, body mass index; FEV_1, forced expiratory volume in 1 second; FVC, forced vital capacity; SD, standard deviation; MITT, multiple-inhaler triple therapy.

Supplementary Table 3. Treatment patterns for patients who stopped MITT in the first 90 days and in patients who stopped MITT between Days 181 and 364

Treatment	Pre-index					
	Patients who stopped MITT in the first 90 days			Patients who stopped MITT between Days 181 and 364		
	Total N=142	Stepping down from MITT N=121	Discontinuing MITT N=21	Total N=162	Stepping down from MITT N=125	Discontinuing MITT N=37
ICS/LABA[1]	92 (64.7)	88 (72.7)	4 (19.1)	111 (68.5)	94 (75.2)	17 (45.9)
No treatment	21 (14.8)	12 (9.9)	9 (42.9)	28 (17.3)	12 (9.6)	16 (43.2)
SABA or SAMA	7 (4.9)	4 (3.3)	3 (14.3)	5 (3.1)	5 (4.0)	0 (0.0)
Other[2]	22 (15.4)	17 (14.0)	5 (24.0)	18 (11.0)	14 (11.2)	4 (10.8)
	Post-index					
ICS/LABA[1]	108 (75.9)	108 (89.3)	0 (0.0)	105 (64.9)	105 (84.0)	0 (0.0)
No treatment	9 (6.3)	0 (0.0)	9 (42.9)	28 (17.3)	0 (0.0)	28 (75.7)
SABA or SAMA	9 (6.3)	0 (0.0)	9 (42.9)	4 (2.5)	0 (0.0)	4 (10.8)
Other[2]	16 (11.2)	13 (10.8)	3 (14.3)	25 (15.5)	20 (16.0)	5 (13.5)

Percentages may not total 100% due to rounding.
[1]ICS/LABA category includes: ICS/LABA, ICS/LABA/SABA or SAMA, ICS/LABA/anti-leukotrienes, ICS/LABA/SABA or SAMA/anti-leukotrienes, ICS/LABA/OCS, ICS/LABA/OCS/SABA or SAMA, ICS/LABA/anti-leukotrienes/OCS; [2]other category includes: ICS, ICS/LAMA, ICS/anti-leukotrienes, ICS/OCS, ICS/OCS/LAMA, LABA/LAMA, LABA/anti-leukotrienes, LAMA, LAMA/anti-leukotrienes, anti-leukotrienes, OCS, OCS/LAMA, OCS/LAMA/anti-leukotrienes.

ICS, inhaled corticosteroids; LABA, long-acting β_2-agonist; LAMA, long-acting muscarinic antagonist; MITT, multiple-inhaler triple therapy; SABA, short-acting β_2-agonist; SAMA, short-acting muscarinic antagonists; OCS, oral corticosteroid.

Supplementary Table 4. Characteristics of patients adherent or non-adherent to MITT in the 12 months following MITT initiation

	**Adherent patients (N=196)**	**Non-adherent patients (N=1008)**
**Demographics**		
Female, n (%)	133 (67.9)	718 (71.2)
Age at asthma diagnosis, mean (SD)	51.2 (15.1)	44.1 (15.8)
Age of MITT initiation, mean (SD)	60.5 (11.9)	53.7 (13.8)
Years from diagnosis to MITT, mean (SD)	9.4 (9.5)	9.6 (8.8)
Urban, n (%)	157 (80.1)	866 (85.9)
Rural, n (%)	39 (19.9)	142 (14.1)
**Smoking status**		
Non-smoker, n (%)[1]	109 (55.6)	599 (59.4)
Smoker, n (%)	38 (19.4)	170 (16.9)
Former smoker, n (%)	45 (23)	202 (20.0)
Unknown, n (%)	4 (2.0)	37 (3.7)
**BMI[2]**	**n=121**	**n=519**
BMI, mean (SD) kg/m^2	30.3 (6.3)	30.2 (6.2)
**Spirometry[3]**	**n=74**	**n=313**
FEV_1 % predicted value, mean (SD)	72.7 (20.2)	70.3 (18.8)
FEV_1/FVC ratio, mean (SD)	71.2 (11.1)	69.9 (13.5)
**Common comorbidities**		
Anxiety/depression, n (%)	80 (40.8)	379 (37.6)
Pneumonia, n (%)	7 (3.6)	50 (5.0)
Other respiratory infections, n (%)	61 (31.1)	312 (31)
Rhinitis, n (%)	35 (17.9)	197 (19.5)
Hypertension, n (%)	91 (46.4)	318 (31.5)
Diabetes mellitus, n (%)	31 (15.8)	108 (10.7)
Gastroesophageal reflux, n (%)	20 (10.2)	92 (9.1)
Conjunctivitis, n (%)	22 (11.2)	99 (9.8)
Polyposis, n (%)	8 (4.1)	68 (6.7)
**Severe exacerbations[4]; n (%)**	113 (57.7)	554 (55.0)
**Blood eosinophils (%), mean (SD)**	**n=119**<br>3.8 (3.5)	**n=613**<br>4.1 (3.4)

[1]The number of non-smokers is higher following MITT as for few patients, the smoking status only became available at that period; [2]reported for patients with BMI record in their medical history; [3]reported for patients with spirometry measurements in their medical history; [4]a severe exacerbation was defined as the need for one hospitalisation or emergency visit or the use of oral/systemic corticosteroids (or increase in the maintenance dose) for ≥3 days due to asthma.

BMI, body mass index; FEV_1, forced expiratory volume in 1 second; FVC, forced vital capacity; MITT, multiple inhaler triple therapy; SD, standard deviation.

Supplementary Table 5. Independent factors associated with discontinuation of MITT determined by the logistic regression model

Variable and reference arm	Estimated adjusted OR[1]	95% CI	p-value in the model
**Sex: male vs female**	1.798	1.106–2.924	0.018
**Previous allergy test: yes vs no**	0.315	0.104–0.950	0.040
**Previous treatment: no vs yes**	3.020	1.497–6.094	0.002

[1]Higher OR is associated with a greater likelihood of discontinuing MITT. Independent variables were age, sex, BMI, smoking status, comorbidities (pneumonia, sleep apnea syndrome, polyposis, allergic rhinitis, atopic dermatitis, conjunctivitis, gastroesophageal reflux, diabetes mellitus, hypertension, anxiety, depression), exacerbations, eosinophil count, chest x-ray, computerized tomography scan, allergy test, GP, nurse and pulmonologist visits, pneumologist and allergy referrals, sick leave, sick leave due to a respiratory cause, influenza and pneumococcal vaccines, and previous treatment (ICS, ICS/LABA, ICS/LABA/anti-leukotrienes, ICS/LABA/OCS/anti-leukotrienes, ICS/LAMA, LAMA, LABA/LAMA, LAMA/anti-leukotrienes, anti-leukotrienes, OCS, no treatment).

BMI, body mass index; CI, confidence interval; GP, general practitioner; ICS, inhaled corticosteroids; LABA, long-acting β_2-agonist; LAMA, long-acting muscarinic antagonist; MITT, multiple-inhaler triple therapy; OCS, oral corticosteroids; OR, odds ratio.
